# Facilitation by *Haloxylon persicum* Shrubs Enhances Density and Richness of Soil Seed Bank of Annual Plants in a Hyper-Arid Ecosystem

**DOI:** 10.3390/plants12061276

**Published:** 2023-03-10

**Authors:** Nasr H. Gomaa, Ahmad K. Hegazy, Haifa Abdulaziz S. Alhaithloul

**Affiliations:** 1 Department of Botany and Microbiology, Faculty of Science, Beni-Suef University, Beni-Suef 62521, Egypt; 2 Department of Botany and Microbiology, Faculty of Science, Cairo University, Giza 12613, Egypt; 3 Biology Department, College of Science, Jouf University, P.O. Box 2014, Sakaka 72388, Saudi Arabia

**Keywords:** annuals, desert, facilitation, seed bank, rainfall

## Abstract

The soil seed bank is an essential functional component of plant communities. In arid ecosystems, the island-like distribution of shrubs influences the spatial distribution of the soil seed bank. Very little is known about seed banks in deserts of the Middle East. The present study aimed to evaluate the facilitative effects of *Haloxylon persicum* shrubs on the soil seed bank of annual plants in a sandy desert region in northwestern Saudi Arabia during two consecutive growing seasons (2017–2018 and 2018–2019) with contrasting rainfall. A total of 480 soil samples at 12 stands were collected from two microhabitats, under shrubs and in open areas, soon after the two growing seasons. The germinable seed bank of annual plants was estimated by controlled seedling emergence method. Shrubs significantly facilitated the accumulation of seed bank beneath their canopies after the two growing seasons. In both microhabitats, the size and species richness of soil seed bank were significantly greater after the wet growing season (2018–2019) than following the dry season (2017–2018). The facilitative effects of shrubs were greater following the moister growing season than after the dry season. The effect of shrubs on seed bank–annual vegetation similarity varied between growing seasons, being greater in shrub interspaces than beneath shrub canopies for the dry growing season, while during the wet season, the similarity of the seed bank with standing annual vegetation was greater in sub-canopy microhabitat than in bare soil.

## 1. Introduction

The seed bank is the viable seeds stored in soil and are available for potential germination and recruitment of new individual plants [1]. Seed banks sustain the diversity of plant communities and contribute to re-colonization [2] and restoration of habitats [3]. Therefore, they have both ecological and applied benefits. Understanding the factors affecting the composition and dynamics of a seed bank will give insights into ecosystem functioning and the future community responses to climate change [4]. Seed banks are very important for population persistence in arid ecosystems where opportunities for seed germination and seedling establishment are unpredictable [5] because rainfall, the major limiting factor, is scarce, unpredictable and highly variable in both space and time [6]. Seed banks allow plants to persist in the environment, enduring successive years without rainfall until appropriate conditions for their germination and establishment occur [7]. Seed banks in desert ecosystems are crucial to maintain the occurrence and genetic diversity of annual plant populations [8,9] because the only way for annual plants to survive in such an unpredictable risky environment is to accumulate a persistent seed bank [10]. Generally, seeds of annual plants constitute a large proportion of seed banks in desert habitats [11,12]. Despite the importance of seed banks in desert ecosystems, studies aiming to understand their composition, spatial and temporal patterns are scarce [4], in particular for the deserts of the Middle East [12].

The size and species composition of seed banks in desert soils are influenced mainly by rainfall, which determines the growth and reproduction of the above-ground vegetation, and thus affects seed yields [13]. Annual variation in rainfall may affect the germination events, growth and seed production of plants [14], and also affect the accumulation of seeds in soil [15,16]. Furthermore, microtopographic conditions indirectly determine the spatial patterns of soil seed banks through driving the distribution of the above-ground vegetation [12].

In addition to physical factors, above-ground vegetation composition influences soil seed reserves in deserts. Plants belonging to different plant life forms with various reproductive strategies contribute differently to the abundance and species richness of the soil seed bank. Trees and shrubs contribute less importantly to seed reserves in soil, while annual species contribute more to soil seed banks than other life forms [12].

Desert vegetation is largely distributed in patches within a bare soil matrix [17]. These patches are usually dominated by shrubs [17] that play a strong nurse role in facilitating their under-canopy plants by creating more favorable microhabitats through mechanisms such as shading, increasing soil moisture content and buffering against extreme temperatures [18,19]. Various studies in arid regions showed that the frequency and intensity of facilitation increase with decreasing rainfall [20,21]. However, some recent studies indicated that facilitative effects of shrubs on their understory plants does not increase monotonically with decreasing precipitation, and that the positive effects of shrubs decrease or cease or may even be reversed under severely low rainfall conditions [22,23]. To interpret this later pattern, these studies suggested that facilitative effects of shrubs on soil moisture diminish [22] or shift to negative influences [23,24] under severely low rainfall deficit.

The differences in habitat conditions between vegetation patches and bare ground affect the overall community composition and structure, and the spatial distribution of seed reserves in soil [25,26]. Shrubs facilitate the accumulation of seeds beneath their canopies by trapping seeds or acting as a barrier for their movement [27]. Shrubs can also indirectly facilitate seed accumulation by acting as a perching site for seed-carrying birds [28] or as a cache for granivorous rodents [29]. Moreover, shrubs may indirectly enhance soil seed banks by facilitating the growth and seed production of the standing annual plant community [26]. Accumulation of seeds under shrubs leads to spatial heterogeneity within soil seed banks, and causes greater seed abundance and seed species diversity beneath shrubs compared to open spaces [30].

The variation in the effects of shrubs on soil moisture with different rainfall amounts may in turn influence the species richness, abundance, growth and reproduction of above-ground plants [22,23,31] and, consequently, soil seed replenishment [8]. Similarly, the individual and interactive effects of shrubs and rainfall on soil moisture content may influence the ability of an above-ground plant community to regenerate from the soil seed bank, which finally determines the seed bank–vegetation similarity, particularly in arid environments where annual plants constitute the main component of both the vegetation and soil seed bank.

The present study aimed to assess the effects of the desert shrub *Haloxylon persicum* Bunge ex Boiss. & Buhse (Chenopodiaceae) on the composition and species richness of soil seed banks of annual plants in northwestern Saudi Arabia during two consecutive growing seasons (2017–2018 and 2018–2019), which differed in precipitation. We hypothesized that: (1) shrubs facilitate the accumulation of soil seed banks, (2) the strength of the facilitative effects would be lower following a dry growing season than after a moister growing season, and (3) shrubs affect the degree of similarity between soil seed banks and the standing vegetation, and their effects vary with growing-season rainfall, with similarity being lower under shrubs than in openings after a dry growing season, but following a wetter season an opposite pattern is predicted.

## 2. Results

**Figure 1:**
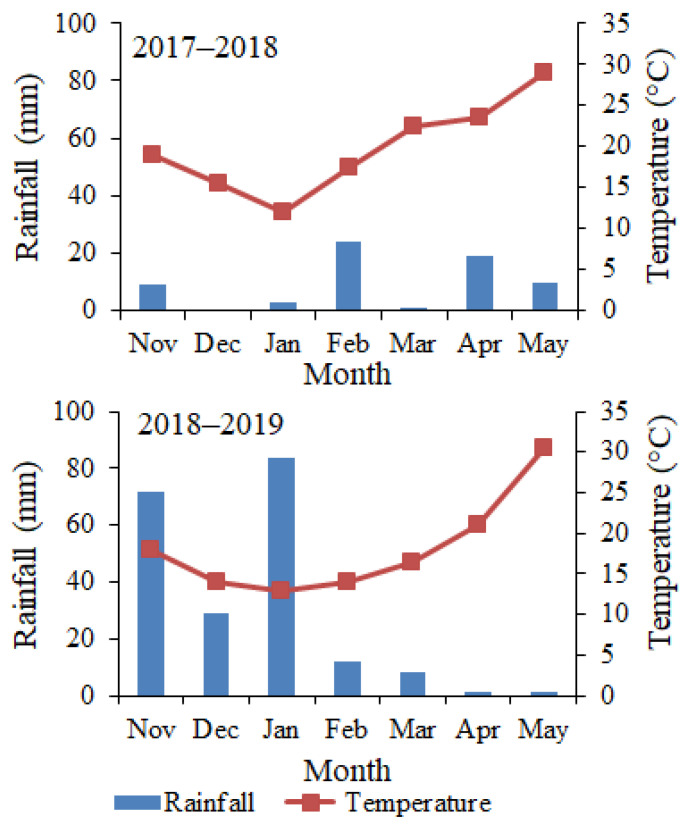
Monthly rainfall and air temperature at the study area during two growing seasons, 2017–2018 and 2018–2019.

The 2017–2018 growing season was dry (64.3 mm), while 2018–2019 was a relatively wet season (207.3 mm). During the first growing season, rains were mainly concentrated in the second half of the season, whereas during 2018–2019, most rains occurred early in the growing season. The patterns of mean monthly air temperature were nearly similar during the two growing seasons (Figure 1).

### 2.1. Seed Bank Composition

In total, 44 annual species belonging to 39 genera were germinated from soil samples over the two growing seasons (Table A1). One of these species, *Anastatica hierochuntica* emerged exclusively from soil collected after the first dry growing season (2017–2018), while other four species, *Anisosciadium lanatum*, *Cleome amblyocarpa*, *Mesembryanthemum nodiflorum* and *Opophytum forsskalii*, were exclusive for soil samples collected after the second growing season (2018–2019). For both seasons, these exclusive plants were recorded from soil collected beneath *H. persicum* shrub canopies. The most abundant annual plants in the germinable seed bank were *Schismus barbatus*, *Eremobium aegyptiacum*, *Bassia muricata*, *Plantago boissieri* and *Cakile arabica* (Table A1).

### 2.2. Shrub Effects on the Soil Seed Bank of Annual Plants

Shrubs of *H. persicum* significantly facilitated the accumulation of soil seed bank of 18 annual species out of 43 and 22 species out of 40 species under their canopies after the 2017–2018 and 2018–2019 growing seasons, respectively. Shrubs did not exert any negative effect on the soil seed bank of annual plants (Table A1).

**Figure 2:**
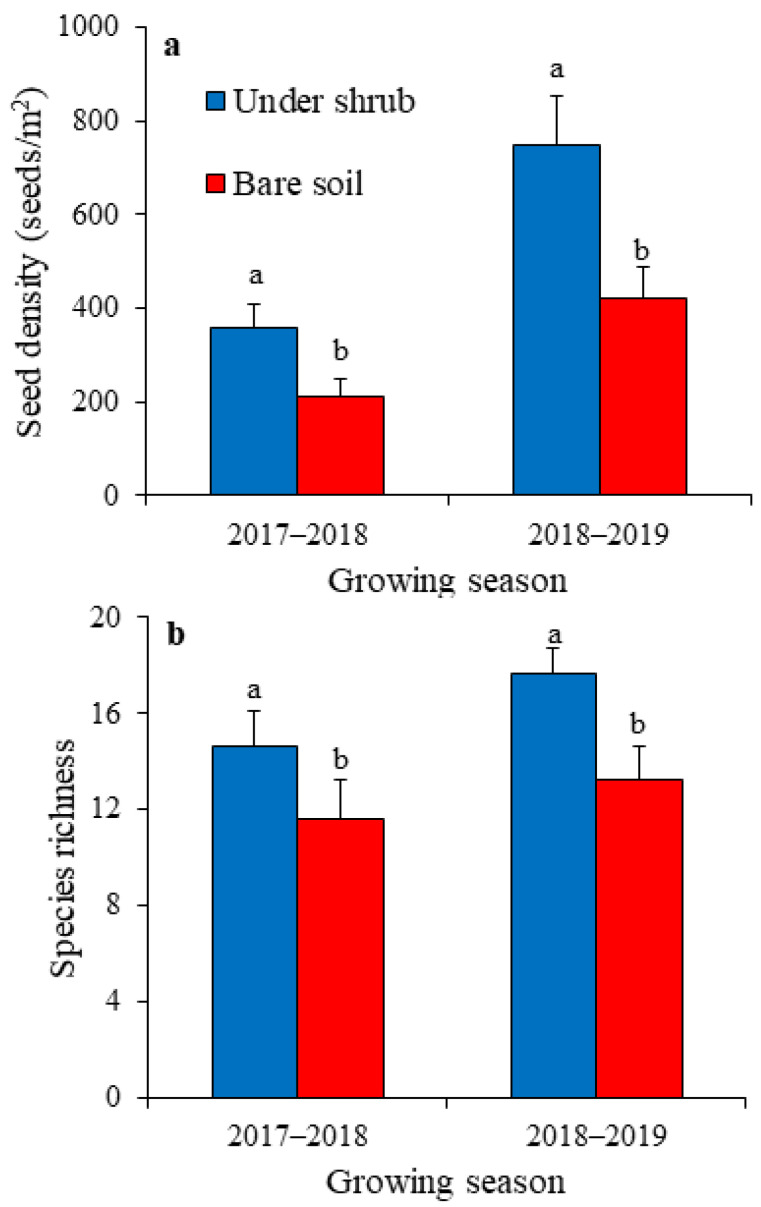
Density (**a**) and species richness (**b**) of germinable soil seed bank of annual plants under shrubs of *Haloxylon persicum* and in bare soil after two growing seasons. Values are means ± SD. Different letters indicate significant difference at *p* < 0.05 between under-shrub and open area after each growing season.

Shrubs and growing seasons significantly influenced the total size (GLM, F = 134.563 and 219.466, respectively, *p* < 0.001) and species richness of germinable soil seed bank (GLM, F = 87.870 and 34.020, respectively, *p* < 0.001). Moreover, shrub × growing season interaction was significant only for species richness (GLM, F = 19.624, *p* < 0.001) (Table 1). After both growing seasons, the total density and species richness of soil seed bank were significantly greater beneath shrubs than in bare ground (Figure 2). The total size of soil seed bank ranged between low values of 213 and 358 seeds/m^2^ after 2017–2018 to high values of 423 and 748 seeds/m^2^ after 2018–2019, for shrub interspaces and sub-canopy microhabitats, respectively (Figure 2a). The species richness of seed bank showed a similar pattern with greater values of 17.7 and 13.3 recorded for sub-canopy and in bare soil microhabitats, respectively, after 2018–2019, and low values following 2017–2018 (14.7 and 11.6 for under shrubs and in open area, respectively) (Figure 2b).

As indicated by the values of the mean relative interaction index (RII) (Table 2), *H. persicum* shrubs significantly enhanced the total density and species richness of the soil seed bank and their facilitative role was greater after the wet growing season (2018–2019) compared with the dry one (2017–2018) (*t*-test, *p* < 0.05).

### 2.3. Shrub Effects on Above-Ground Annual Vegetation and Seed Bank–Vegetation Similarity

**Figure 3:**
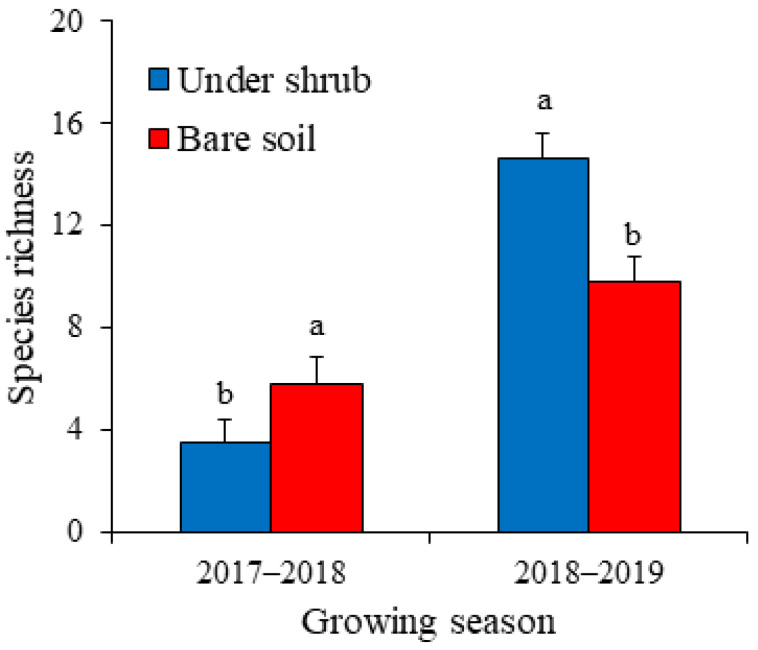
Species richness of standing annual vegetation under shrubs of *Haloxylon persicum* and in bare soil for two growing seasons. Values are means ± SD. Different letters indicate significant difference at *p* < 0.05 between under-shrub and open area in each growing season.

**Figure 4:**
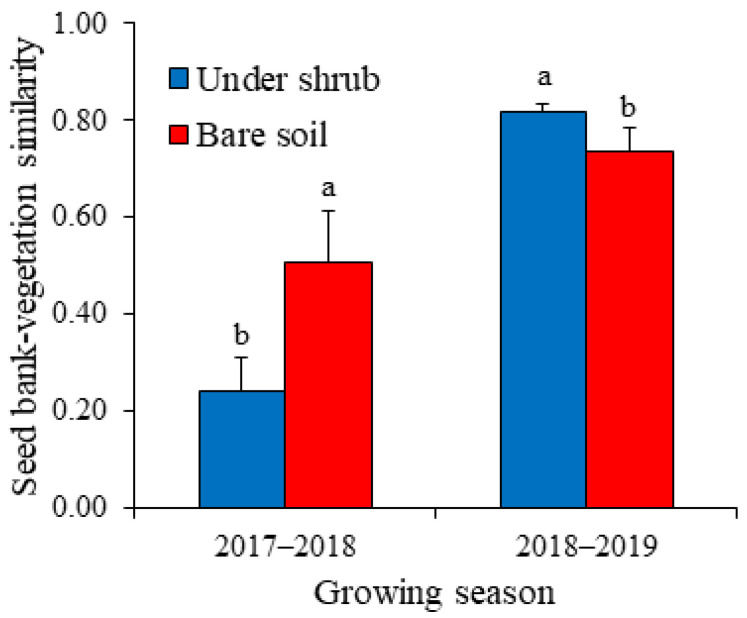
Seed bank–vegetation similarity for annual plants under shrubs of *Haloxylon persicum* and in bare soil for two growing seasons. Values are means ± SD. Different letters indicate significant difference at *p* < 0.05 between under-shrub and open area for each growing season.

The species richness of the standing annual vegetation and seed bank–vegetation similarity were significantly affected by shrub and growing season (GLM, *p* < 0.001). The interactive effect of shrub and growing season was significant for the species richness of the standing vegetation and the similarity between seed bank and above-ground vegetation (Table 3) (GLM, F = 147.559 and 71.490, respectively, *p* < 0.001). The species richness of the above-ground annual vegetation was significantly greater in bare soil (5.8) than in sub-canopy areas (3.5) during the dry 2017–2018 season, whereas in 2018–2019 the species richness was greater under shrubs (14.6) compared with open area (9.8) (Figure 3). Similar to the pattern of species richness of the standing annual vegetation, seed bank–vegetation similarity was greater in shrub interspaces (0.51) than beneath shrub canopies (0.24) for 2017–2018, while for the wet growing season (2018–2019), the similarity of soil seed bank with standing annual vegetation was greater in sub-canopy microhabitat (0.82) than in bare soil (0.73) (Figure 4). Values of the mean relative interaction index were significantly negative (–0.25 ± 0.06) for species richness of the above-ground annual vegetation during 2017–2018, while in 2018–2019 the RII values were significantly positive (0.20 ± 0.03). This means that *H. persicum* shrubs facilitated the understory annual plants during the wet growing season, while in the dry season they exerted negative effects.

### 2.4. Shrub Effects on Soil Properties

The effect of *H. persicum* shrubs was significant on all the measured soil parameters, whereas the growing season effect was significant only for soil moisture content (GLM, F = 1867.863, *p* < 0.001). None of the shrub × growing season interactions were significant except for moisture content (GLM, F = 26.047, *p* < 0.001) (Table 4). The soil parameters, organic carbon, electrical conductivity, silt + clay, N, P, and K, were significantly greater beneath shrubs than in bare ground. By contrast, sand % was significantly lower in sub-canopy microhabitat compared with open area (Table 5). Soil moisture content was significantly lower beneath shrubs than in bare ground during the dry 2017–2018 season, while in the wet season (2018–2019) an opposite pattern was observed (Table 5).

## 3. Discussion

The total density of germinable soil seed bank of annual plants in the study area is relatively low, varying between 213 and 748 seeds/m^2^. Comparable low densities of germinable seed bank of annual species were reported in the sandy Monte Desert in Argentina [15], where seed density ranged between 295.2 and 608.1 seeds/m^2^. Most studies on soil seed banks, particularly in arid regions, were based on the total direct seed counts from soil samples and not on the germinable fraction of seeds, resulting in higher seed estimations [32].

Our results support the hypothesis that shrubs accumulate a large and diverse seed bank beneath their canopies. Accumulation of seed bank under shrubs may be the result of seed input received by wind and water and trapped beneath shrubs [27,33]. Shrubs also offer perches and food to birds, which may contribute to recruiting zoochorous species to soil seed banks [34]. By modifying the physical and chemical properties of soil, improving soil microrelief, reducing direct sunlight, and enhancing soil moisture and fertility, shrubs can indirectly increase the soil seed bank by facilitating colonization, growth, flowering, and seeding of herbaceous species under their canopies [18,19]. In the present study, *H. persicum* shrubs improved sub-canopy soil fertility by enhancing soil organic carbon and nutrients (N, P and K). Compared to bare ground, soils beneath *H. persicum* canopies had greater content of silt and clay, which may be related to the accumulation of wind-blown, fine soil particles below shrubs [35]. The fine soil texture under shrubs facilitates the accumulation of more seeds in the soil seed bank [36]. It is apparent that shrubs affect the soil seed bank through various mechanisms. In spite of not knowing the relative contribution of each of these mechanisms in determining the impact of *H. persicum* shrubs on the soil seed bank, we suggest that the physical role of shrubs in trapping seeds is the most important, followed by the facilitative effects of shrubs on above-ground vegetation.

For both microhabitats, the density of germinable soil seed bank was about twice larger after the wet growing season than after the dry season. Likewise, the species richness of the seed bank was about 1.2 times greater following the wet growing season than after the dry season. The enhancing effects of rainfall on seed banks were reported by Gutiérrez and Meserve [32] in an arid thorn scrub community in north-central Chile, Li et al. [37] in the Hengduan Mountains region of southwest China, and Gomaa [16] in the Eastern Desert of Egypt.

In accordance with our hypothesis, the facilitative effects of *H. persicum* shrubs on the soil seed bank were more intense after the wet growing season than after the dry season. This may be attributed to the variation in the effects of shrubs on the above-ground annual vegetation between growing seasons, where shrubs enhanced the standing annual plants, as measured by species richness, during the wet growing season, consequently increasing seed output and enriching the soil seed bank. Conversely, shrubs exerted a negative effect on the understory above-ground annual species during the dry season, which finally reduces seed yield. The change in the effect of shrubs on annual vegetation between the two growing seasons in our study may be related to the fact that shrubs enhanced water availability in the sub-canopy soil during the wet growing season, but reduced soil moisture content beneath their crowns during the relatively dry season. Similar observations were reported by O’Brien et al. [24] in a semi-arid shrubland and by Gomaa et al. [23] in a hyper-arid Arabian desert. During small rain events, shrubs might decrease water availability beneath their canopies by intercepting rainwater [31,38]. Additionally, water intercepted by shrub crowns during small rain events is lost to evaporation and in wetting the canopy surface, and is less likely to reach soil below shrubs [39]. Under severely low rainfall, all of these factors make the sub-canopy soil dryer than soil of the shrub interspaces. Conversely, under moderate and heavy rains, shrubs pass water intercepted by their crown to the understory soil through stemflow [40,41]. In addition, the low evaporation under shrubs [22] may increase moisture retention in the sub-canopy soil. Therefore, during a relatively wet year, sub-canopy soil is wetter than bare soil.

There is a correspondence between the patterns of species richness of annual vegetation and seed bank–vegetation similarity. For both microhabitats, the overall similarity between seed bank and standing annual vegetation was greater in the wet growing season than in the dry season. At microhabitat level, the similarity was greater in open areas than under shrubs in the dry season. By contrast, the soil seed bank was more similar to above-ground vegetation in sub-canopy microhabitat than in bare ground during the moist growing season. These results suggest that the greater the species richness of the above-ground annual vegetation, the greater the degree of similarity between the floristic composition of the seed bank and the standing annual vegetation. Communities characterized by preponderance of annual plants show high resemblance between the seed bank and vegetation composition [12,42] because annual plants rely mainly on the seed bank for their regeneration and contribute more to the seed bank than perennials. The degree of correspondence between soil seed bank and standing annual vegetation recorded in our study was relatively high (overall similarity mean = 0.58) and comparable to that reported for the north edge of the Taklimakan Desert [43], southern Gurbantunggut Desert dunes during winter [44] and sandy grasslands of eastern Inner Mongolia [45], where the similarity coefficients were 0.778, 0.63 and 0.66, respectively.

During the wet growing season, which had the greatest species richness of both the seed bank and above-ground annual plants, the average seed bank–vegetation similarity was high (>0.70). This suggests the potential use of soil seed banks for the restoration of annual vegetation in case of habitat deterioration due to anthropogenic activities or climate change effects. Our results showed also that *H. persicum* shrubs enhanced soil fine particle content, soil nutrients and accumulation of the soil seed bank beneath their crowns. Therefore, *H. persicum* shrubs could be applied in the rehabilitation of desertified ecosystems in arid regions. Desert shrubs are known as potential tools in the restoration of desertified arid lands [46].

## 4. Materials and Methods

### 4.1. Study Area

**Figure 5:**
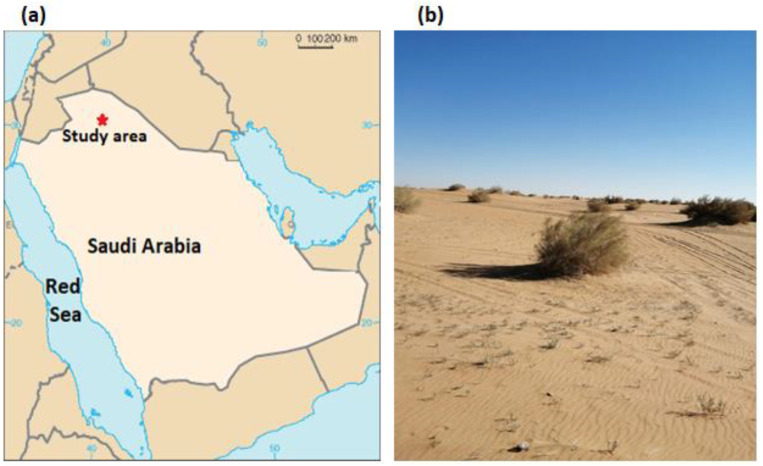
A map showing the location of the study area (**a**); a community dominated by *Haloxylon persicum* (**b**).

The study area is within sandy desert, located at the northern reaches of the Nafud Desert, the large sand-dune desert in Saudi Arabia (Figure 5a). The study region is located some 15 km south of Dumat al-Jandal city (29°48′41″ N, 39°52′6″ E) in Al-Jouf Region, northwestern Saudi Arabia. The climate is hyper-arid with 59 mm mean annual precipitation. Rainfall varies in timing and amount, and occurs mainly between November and May. The area is characterized by hot summers and cool winters. The average monthly air temperature varied between a low of 9.8 °C in January and a high of 33.8 °C in August. The lowest (16%) and highest (53%) mean monthly relative humidity were recorded in June and in January, respectively.

The study region is dominated by widely spaced shrubs of *H. persicum* (Figure 5b). Annual plants grow during brief periods of relatively abundant moisture beneath shrubs and in the interspaces. The common co-occurring perennial species include *Haloxylon salicornicum* (Moq.) Bunge ex Boiss. (Chenopodiaceae), *Nauplius graveolens* (Forssk.) Wiklund (Asteraceae) and *Artemisia judaica* L. (Asteraceae).

*Ghada* shrubland dominated by *H. persicum* spreads over thousands of square kilometers in the great deserts of eastern Arabia [47]. The geographical distribution of *H. persicum* covers several regions, including in Central Asia, the Middle East, Afghanistan, northwestern China and the Near Eastern deserts [48,49]. *H. persicum* is very tolerant to environmental extremes in temperature and water deficit [50]. It has both economic and ecological benefits in arid zones because it plays vital roles in stabilizing sand dunes, conserving soil and water, reducing desertification rate [51] and enhancing biodiversity by improving environmental conditions and providing shelter for many other plant associations [52]. The plant is also a potential source of firewood and used in furniture, paper, and dye manufacturing [53].

### 4.2. Shrub Effects on Above-Ground Annual Plants

To assess the effects of *H. persicum* shrubs on the above-ground annual plants, which may indirectly influence the soil seed bank, we measured the species richness of the standing annual vegetation in response to shrub occurrence. A total of 12 stands (40 m × 40 m each) were selected at the study area so as to include all variations in the annual vegetation within the communities dominated by *H. persicum* at the study area. At every stand, 10 *H. persicum* shrubs and 10 open areas nearby were chosen randomly. A quadrat (1 m × 1 m) was placed randomly below each selected shrub and a similar quadrat was laid randomly in the bare ground adjacent to the selected shrub. The bare ground microhabitat was at least 2 m away from the canopy edge of any shrub. A list of the annual species present in the quadrats was compiled in March during the two growing seasons, 2017–2018 and 2018–2019. The growing season coincides with the rainy period and extends from November to May. The species richness of the standing annual vegetation was determined as the number of species per microhabitat (under shrubs or bare soil) per stand. Species identification and nomenclature followed Chaudhary [54,55,56].

### 4.3. Shrub Effects on the Soil Seed Bank of Annual Plants

Soil samples were collected from the selected 12 stands in the study area after seed dispersal in early June under shrubs of *H. persicum* and in open areas following the two growing seasons, 2017–2018 and 2018–2019. For each stand, 10 samples, each 25 cm × 20 cm and 5 cm depth, were randomly taken from soil beneath the selected 10 *H. persicum* shrubs, and another 10 soil samples of the same volume were collected from the 10 open areas. The samples were sieved through a 4 mm sieve to remove plant fragments and coarse stones. Known volumes of sieved soil samples (500 cm^3^) were sown in 25 cm × 20 cm × 8 cm germination trays and regularly irrigated every two days in a greenhouse (29°54′40.9″ N 39°46′41″ E; 670 m.a.s.l.) located about 30 km away from the study area. Emerging seedlings were identified, counted and removed. After six months (November–April) the experiment was stopped, because no more seedlings appeared for three consecutive weeks. For every microhabitat (beneath shrub and open area) per stand per growing season, a floristic list was compiled and the mean number of germinable seeds per m^2^ for each species was determined. Moreover, the total density of germinable seed bank was estimated as the number of seeds of all species per m^2^, and the species richness of the seed bank was measured as the total number of species present.

### 4.4. Soil Analysis

Three soil samples (0–30 cm depth) were taken randomly from every microhabitat per stand in March of the two growing seasons. The percentage of sand (>0.05 mm) and silt + clay (<0.05 mm) was estimated by sieving 100 g of soil sample through a 0.05 mm sieve. Electrical conductivity of soil–water extract (1:5 *w*/*v*) was measured using an electrical conductivity meter. Oxidizable soil organic carbon was determined by the Walkley and Black procedure. Soil moisture content was evaluated by drying soil in an oven at 105 °C for 48 h. Available nitrogen was estimated by the micro-Kjeldahl method, while available phosphorus was measured by the Olsen method, using sodium bicarbonate as an extracting agent. The available potassium content was determined using a flame photometer. All soil analyses were performed according to methods described by Black [57] and Gupta [58].

### 4.5. Data Analysis

A general linear model (GLM) was applied to test the effects of two main factors, (A) shrub: (1) under-shrub and (2) shrub interspaces; and (B) growing season: (1) 2017–2018 and (2) 2018–2019, on the total density and species richness of the soil seed bank of annual plants, species richness of the above-ground annual vegetation, seed bank–annual vegetation similarity, and soil properties. Relative interaction indices (RII) [59] were applied to evaluate the influence of *H. persicum* shrubs on four community attributes: the density of seed bank for individual species, the total density and species richness of the seed bank, and the species richness of the standing annual vegetation. RII = (CAu − CAo)/(CAu + CAo), where CAu and CAo are the community attributes for under-shrub and in the open area next to it, respectively; RII values range from −1 to +1. Negative values show negative effects of shrubs on the seed bank parameter, positive values indicate facilitative effects, and a zero value displays a neutral effect. The one-sample *t*-test was applied to check whether RII values differ significantly from zero. In order to assess the change in the strength of the facilitative effect of shrubs on germinable soil seed bank of annual species between the two growing seasons, we used the independent samples *t*-test to compare between the relative interaction indices of the two growing seasons. The same test was also used to compare between the values for the measured seed bank, vegetation and soil parameters of sub-canopy microhabitat versus shrub interspaces. The general linear model and *t*-tests were applied using SPSS v.16 software (SPSS, Chicago, IL, USA).

## 5. Conclusions

*H. persicum* shrubs and growing season rainfall influenced the floristic composition and diversity of the germinable soil seed banks of annual plants. In addition to their roles in trapping seeds, shrubs may indirectly influence the soil seed bank through their effects on above-ground vegetation. *H. persicum* shrubs enhanced the total size and species richness of the soil seed bank and their facilitative effects were greater after a moister growing season than following a drier season. In both open area and sub-canopy microhabitats, the density and species richness of the soil seed bank were greater after the wet growing season than following the dry season. The effect of shrubs on the degree of resemblance between the seed bank and above-ground annual vegetation varied with growing season rainfall, being greater in open areas than under shrubs for the dry growing season, while for the moister season, the opposite held true. The variation in the effects of shrubs on seed bank parameters between growing seasons may be related to changes in the effects of shrubs on moisture availability with growing season rainfall. Shrubs enhanced soil moisture content beneath their canopies during the wet growing season, whereas during the dry season, they had a negative effect.

## Figures and Tables

**Table 1:** General linear model analysis testing the effects of shrub (under-shrub vs. bare soil) and growing season on the density and species richness of germinable soil seed bank of annual species. F-values are shown.

Factor	Seed Density	Species Richness
Shrub	134.563 ***	87.870 ***
Growing season	219.466 ***	34.020 ***
Shrub × growing season	19.624 ***	2.777 ns

***, *p* < 0.001; ns, non-significant.

**Table 2:** Relative interaction index (RII) values (mean ± SD) showing the effects of *Haloxylon persicum* shrubs on the density and species richness of germinable soil seed bank of annual plants after two growing seasons.

Growing Season	Seed Density	Species Richness
2017–2018	0.26 ± 0.02 *^b^	0.12 ± 0.03 *^b^
2018–2019	0.28 ± 0.02 *^a^	0.14 ± 0.03 *^a^

*, RII values differ significantly from 0 at *p* < 0.05 based on one-sample *t*-test; means with different letters in a column show significant difference between the two growing seasons at *p* < 0.05 according to *t*-test.

**Table 3:** General linear model analysis testing the effects of shrub (under-shrub vs. bare soil) and growing season on species richness of the standing annual vegetation and seed bank–annual vegetation similarity. F-values are shown.

Factor	Species Richness	Seed Bank–Vegetation Similarity
Shrub	18.821 ***	20.102 ***
Growing season	692.707 ***	387.073 ***
Shrub × growing season	147.559 ***	71.490 ***

***, *p* < 0.001.

**Table 4:** General linear model testing the effects of shrub (under-shrub vs. bare soil) and growing season on soil properties. F-values are shown.

Parameter	Shrub	Growing Season	Shrub × Growing Season
Organic carbon (%)	18.160 ***	0.077 ns	0.009 ns
Moisture content (%)	7.430 **	1867.863 ***	26.047 ***
Electrical conductivity (dS/m)	155.033 ***	1.948 ns	0.007 ns
Sand	634.707 ***	0.036 ns	0.024 ns
Silt + clay	634.707 ***	0.036 ns	0.024 ns
N (mg/kg)	33.869 ***	0.006 ns	0.024 ns
P (mg/kg)	815.276 ***	0.002 ns	0.143 ns
K (mg/kg)	242.818 ***	0.005 ns	0.053 ns

***, *p* < 0.001; **, *p* < 0.01; ns, non-significant.

**Table 5:** Soil properties under shrubs of *Haloxylon persicum* and in bare soil. Values are means ± SD. Microhabitats in a given growing season sharing the same letter are not significantly different at *p* < 0.05 according to independent samples *t*-test.

Soil Variable	2017–2018		2018–2019	
	Under Shrub	Bare Soil	Under Shrub	Bare Soil
Organic carbon (%)	0.31 ± 0.08 ^a^	0.22 ± 0.08 ^b^	0.30 ± 0.08 ^a^	0.21 ± 0.08 ^b^
Moisture content (%)	1.15 ± 0.16 ^b^	1.31 ± 0.18 ^a^	4.04 ± 0.32 ^a^	3.87 ± 0.32 ^b^
Electrical conductivity (dS/m)	0.73 ± 0.04 ^a^	0.55 ± 0.07 ^b^	0.71 ± 0.04 ^a^	0.53 ± 0.06 ^b^
Sand (%)	84.4 ± 1.0 ^b^	92.1 ± 0.6 ^a^	84.1 ± 1.6 ^b^	91.8 ± 1.2 ^a^
Silt + clay (%)	15.6 ± 1.0 ^a^	7.9 ± 0.6 ^b^	15.9 ± 1.6 ^a^	8.2 ± 1.2 ^b^
N (mg/kg)	102.7 ± 8.1 ^a^	90.9 ± 7.7 ^b^	103.2 ± 7.0 ^a^	91.4 ± 6.7 ^b^
P (mg/kg)	2.49 ± 0.11 ^a^	1.35 ± 0.16 ^b^	2.47 ± 0.12 ^a^	1.37 ± 0.15 ^b^
K (mg/kg)	117.3 ± 7.6 ^a^	80.4 ± 9.9 ^b^	118.5 ± 6.0 ^a^	79.7 ± 9.8 ^b^

## Data Availability

The data presented in this study are available on request from the corresponding author.

## Appendix A

**Table A1:** Density of the soil seed bank of annual species under shrubs of *Haloxylon persicum* and in bare soil after two growing seasons. Relative interaction index (RII) values are indicated. *, RII values differ significantly (*p* < 0.05) from zero according to one-sample *t*-test; ns, non-significant.

Species	2017–2018			2018–2019		
	Density (Seeds m^−2^)		RII	Density (Seeds m^−2^)		RII
	Under Shrub	Bare Soil		Under Shrub	Bare Soil	
*Agriophyllum minus* Fisch. & C.A.Mey.	6.7	3.3	0.40 ns	14.2	6.7	0.58 *
*Aizoon hispanicum* L.	0.8	0.0	–	0.8	0.0	–
*Anastatica hierochuntica* L.	0.8	0.0	–	0.0	0.0	
*Anisosciadium lanatum* Boiss.	0.0	0.0		0.8	0.0	–
*Anthemis haussknechtii* Boiss. & Reut.	16.7	10.8	0.21 *	35.8	21.7	0.34 *
*Arnebia decumbens* (Vent.) Coss. & Kralik	1.7	0.8	–	1.7	0.8	–
*Asteriscus hierochunticus* (Michon) Wiklund	1.7	0.8	–	4.2	2.5	0.57 ns
*Astragalus arpilobus* Kar. & Kir.	15.0	9.2	0.36 *	32.5	19.2	0.29 *
*Astragalus asterias* Hohen	7.5	3.3	0.37 *	15.8	10.0	0.43 ns
*Bassia eriophora* (Schrad.) Asch.	9.2	4.2	0.53 *	21.7	13.3	0.35 *
*Bassia muricata* (L.) Asch.	31.7	20.8	0.28 *	64.2	36.7	0.32 *
*Brassica tournefortii* Gouan	10.0	6.7	0.22 *	22.5	12.5	0.49 *
*Cakile arabica* Velen. & Bornm.	21.7	14.2	0.23 *	45.8	28.3	0.31 *
*Calendula tripterocarpa* Rupr.	4.1	1.7	0.42 ns	7.5	5.0	0.43 ns
*Cleome amblyocarpa* Barratte & Murb.	0.0	0.0		1.7	0.0	–
*Cutandia memphitica* (Spreng.) K.Richt.	13.3	8.3	0.24 *	26.7	16.7	0.32 *
*Diplotaxis acris* (Forssk.) Boiss.	1.7	0.0	–	6.7	1.7	0.80 *
*Eremobium aegyptiacum* (Spreng.) Asch. & Schweinf. ex Boiss.	30.8	22.5	0.16 *	65.0	38.3	0.26 *
*Erodium laciniatum* (Cav.) Willd.	5.0	2.5	0.47 ns	8.3	3.3	0.53 ns
*Horwoodia dicksoniae* Turrill	2.5	0.8	0.67 ns	3.3	0.0	–
*Ifloga spicata* (Forssk.) Sch. Bip.	18.3	10.8	0.39 *	39.2	22.5	0.31 *
*Limonium lobatum* (L.f.) Chaz.	0.8	0.8	–	0.8	0.0	–
*Malcolmia grandiflora* (Bunge) Kuntze	4.1	1.7	0.42 ns	6.7	4.2	0.24 ns
*Malva parviflora* L.	8.3	5.0	0.33 ns	15.0	9.2	0.26 *
*Matthiola longipetala* (Vent.) DC.	9.2	5.0	0.41 *	15.8	6.7	0.59 *
*Medicago laciniata* (L.) Mill.	0.8	0.0	–	0.8	0.0	–
*Mesembryanthemum nodiflorum* L.	0.0	0.0		0.8	0.0	–
*Neurada procumbens* L.	1.7	0.8	0.50 ns	1.7	0.8	–
*Notoceras bicorne* (Aiton) Amo	1.7	0.8	–	3.3	2.5	–
*Oligomeris linifolia* (Vahl ex Hornem.) J.F.Macbr.	1.7	0.8	–	3.3	2.5	–
*Opophytum forsskalii* (Hochst. ex Boiss.) N.E.Br.	0.0	0.0		0.8	0.0	–
*Paronychia arabica* (L.) DC.	0.8	0.0	–	1.7	0.8	0.50 ns
*Plantago boissieri* Hausskn. & Bornm.	25.8	16.7	0.24 *	50.0	33.3	0.27 *
*Plantago amplexicaulis* Cav.	4.2	2.5	0.33 ns	8.3	3.3	0.57 *
*Plantago ciliata* Desf.	9.2	2.5	0.75 *	19.2	10.0	0.33 *
*Plantago ovata* Forssk.	10.0	4.2	0.60 *	19.2	9.2	0.55 *
*Pteranthus dichotomus* Forssk.	0.8	0.0	–	1.7	0.0	–
*Rumex vesicarius* L.	5.8	0.8	0.87 *	11.7	2.5	0.74 *
*Savignya parviflora* (Delile) Webb	10.8	7.5	0.17 *	25.0	14.2	0.43 *
*Schimpera arabica* Hochst. & Steud. ex Steud.	1.7	0.8	–	5.0	2.5	0.71 ns
*Schismus barbatus* (L.) Thell.	43.3	31.7	0.18 *	89.2	54.2	0.24 *
*Silene arabica* Boiss.	15.8	9.2	0.28 *	35.0	20.8	0.42 *
*Spergularia bocconei* (Scheele) Graebn.	0.8	0.0	–	3.3	0.8	0.67 ns
*Trigonella stellata* Forssk.	4.2	1.7	0.58 ns	11.7	5.0	0.51 *

–, species was excluded from the RII analysis because it was recorded in only one stand in a growing season.
